# Triazoles as a Potential Threat to the Nutritional Quality of Tomato Fruits

**DOI:** 10.3390/metabo13090988

**Published:** 2023-09-01

**Authors:** Veronika Hýsková, Michal Jakl, Jana Jaklová Dytrtová, Sanja Ćavar Zeljković, Ondřej Vrobel, Kateřina Bělonožníková, Daniel Kavan, Tomáš Křížek, Alice Šimonová, Marie Vašková, Ishak Kovač, Antoniana Račko Žufić, Helena Ryšlavá

**Affiliations:** 1 Department of Biochemistry, Faculty of Science, Charles University, Albertov 6, 128 00 Prague, Czech Republic; veronika.hyskova@natur.cuni.cz (V.H.); katerina.belonoznikova@natur.cuni.cz (K.B.); daniel.kavan@natur.cuni.cz (D.K.); ouskovam@natur.cuni.cz (M.V.); zuficnina@gmail.com (A.R.Ž.); 2 Department of Agroenvironmental Chemistry and Plant Nutrition, Faculty of Agrobiology, Food and Natural Resources, Czech University of Life Sciences Prague, Kamýcká 129, 165 00 Prague, Czech Republic; jakl@af.czu.cz; 3 Sport Sciences—Biomedical Department, Faculty of Physical Education and Sport, Charles University, José Martího 269, 162 52 Prague, Czech Republic; dytrtova@ftvs.cuni.cz (J.J.D.); ikovac@ftvs.cuni.cz (I.K.); 4 Centre of the Region Haná for Biotechnological and Agricultural Research, Department of Genetic Resources for Vegetables, Medicinal and Special Plants, Crop Research Institute, Šlechtitelů 241/27, 783 71 Olomouc, Czech Republic; sanja.cavar@upol.cz (S.Ć.Z.); ondrej.vrobel@upol.cz (O.V.); 5 Czech Advanced Technology and Research Institute, Palacký University, Křížkovského 511/8, 779 00 Olomouc, Czech Republic; 6 Department of Analytical Chemistry, Faculty of Science, Charles University Albertov 6, 128 00 Prague, Czech Republic; tomas.krizek@natur.cuni.cz (T.K.); alice.simonova@natur.cuni.cz (A.Š.)

**Keywords:** fruit, tomato, triazoles, penconazole, tebuconazole, stress, nutrition

## Abstract

Triazole fungicides can threaten plants as abiotic stressors but can also positively affect plant defense by inducing priming. Thus, plant yield is also both protected and endangered by triazoles that may influence several metabolic pathways during maturation processes, such as the biosynthesis of saccharides or secondary metabolites. Here, *Solanum lycopersicum* L. plants were exposed to foliar and soil applications of penconazole, tebuconazole, or their combination, and their resulting effect on tomato fruits was followed. The exposure to the equimolar mixture of both triazoles influenced the representation of free proteinogenic amino acids, especially Gln, Glu, Gly, Ile, Lys, Ser and Pro, saccharide content, and led to a significant increase in the contents of total phenolics and flavonoids as well as positive stimulation of the non-enzymatic antioxidant system. Among the identified secondary metabolites, the most abundant was naringenin, followed by chlorogenic acid in tomato peel. In turn, all triazole-treated groups showed a significantly lower content of rosmarinic acid in comparison with the control. Foliar application of penconazole affected the fruit more than other single triazole applications, showing a significant decrease in antioxidant capacity, the total content of secondary metabolites, and the activities of total membrane-bound peroxidases and ascorbate peroxidase.

## 1. Introduction

The fruits of plants are an important component of the human diet. The human digestive system evolved with a significant representation of diverse plant resources in the era of hunters and gatherers, but the transition to agriculture has reduced species diversity and especially increased the representation of starches. Therefore, current modern trends in nutrition recommend increasing the intake of raw fruits and vegetables [1]. Plant fruits contain several valuable, health-promoting substances. These include soluble fiber, which lowers the glycemic index of foods and positively influences the composition of the human microbiome. Fruits also contain saccharides, mainly fructose, which is a source of energy but is present only in quantities that do not adversely affect human physiology. Other phytonutrients, carotenoids, and phenolic compounds such as flavonoids, including anthocyanins, which have antioxidant and anti-inflammatory effects, are important. The intake of these substances means the prevention of cardiovascular diseases, diabetes type II and other diseases of civilization [1,2]

Hydroxycinnamic acid esters are the most abundant phenolic substances in Solanaceae species, especially chlorogenic acid, which has significant antioxidant capacity and hepatoprotective, hypoglycemic and antiviral activities [3]. Tomato fruits are also rich in terpenoids: carotenoids, α- and β-carotene, and lycopene, which, although not a precursor of vitamin A, is an important antioxidant [1,4]. Tomato plants also contain glycoalkaloids, especially α-tomatine, which seems to be toxic to various fungi, insects, animals, and cancer cells. Tomatine and dehydrotomatin are present in green tomato fruits, but during ripening, tomatine is converted to esculeosides via hydroxylation, acetylation and glycosylation. Esculeoside A has health benefits; it inhibits acyl-Co A cholesterol transferase, prevents LDL oxidation, and prevents the conversion of macrophages into foam cells, thus reducing atherosclerosis [3,5]. Anticarcinogenic effects of steroidal glycoalkaloids, including tomatine, have been described [6].

Tomato plants are susceptible to many types of fungi and must therefore be treated with antifungal agents such as biological control agents or triazole fungicides [7,8,9]. Antifungal triazoles, e.g., penconazole and tebuconazole, inhibit the biosynthesis of ergosterol by blocking sterol 14-α-demethylase (CYP51, EC 1.14.14.154), which leads to the disruption of fungal plasma membranes [10,11,12,13]. Triazoles are often applied foliarly and most likely represent the largest and a key group of systemic chemicals developed for the control of fungal and yeast crop diseases. Additionally, triazoles can create various complexes with metal ions, resulting in new features and dangerous impacts on non-target organisms in the environment [8,9,14,15,16,17,18,19]. Although originally used in agriculture, triazoles easily spread through the soil, contaminate groundwater, and even reach urban centers [20]. Fungicides can alter the microbiome of the soil, including the amount and taxonomic variety of bacteria and fungi, including symbiotic and growth-promoting microorganisms [21]. Moreover, they are also used in therapeutic and personal care products and can be found in surface water and aquatic sediment [22,23,24,25]. For example, several developmental and morphological malformations and other adverse effects on non-targets like zebrafish (*Danio rerio*) were reported if exposed to tebuconazole, cyproconazole, clotrimazole and ketoconazole [24,26,27]. Several azoles were shown to be potential endocrine disruptors [11,28,29]. Some triazoles also show both toxic and stimulant effects on plants [30]. Triazoles were shown to stimulate chlorophyll biosynthesis, accelerate chloroplast differentiation, and protect the integrity of chlorophyll [31,32]. The soil application of cyproconazole and penconazole led to a decrease in the green biomass of tomato plants, altered phenolic representation in fruit, increased fruit size and yield, and decreased the mobility of several key elements (Fe, Cu and Zn) [9]. Triazoles can accumulate in fruits and affect their development and nutritional quality, which could be dangerous for consumers [33,34]. In the extensive study of the impact of tebuconazole on the flavor and color components of Merlot and Cabernet Sauvignon wines, differences were mostly found in the fruity and floral characteristics of the wine due to changes in the levels of acids, alcohols and esters [35]. Paclobutrazol showed the greatest influence on wine flavor and aroma composition, especially through changes in the concentration of various esters and acids, followed by tebuconazole and triadimefon [36]. 

The nutritional value of the tomato fruit is determined by the presence of both primary and secondary metabolites. The monitored products of primary metabolism include carbohydrates, free amino acids and proteins. However, the quality of tomato fruit is also determined by the presence of secondary metabolites that have antioxidant capacity and other health benefits. Triazoles are known to affect the activity of cytochrome P450 enzymes and could therefore reduce the synthesis of some phenolic and terpenoid compounds. On the other hand, the fungicides could function as abiotic stress and increase the synthesis of secondary metabolites. 

There is an unpleasant struggle with developing fungal resistances against various fungicides. Fungi have several ways to defend themselves, such as modification of their sensitive sites, exclusion or detoxification of the fungicide [37,38]. Therefore, more and more fungicide mixtures are being used and accumulating in the environment, where they represent an ever-increasing threat to non-target organisms [22]. 

Here, we aimed to analyze the influence of penconazole, tebuconazole or their combination through their foliar and soil applications on the nutritional quality of tomato yield. We tested our hypothesis to see if triazoles may disrupt several biosynthetic pathways in tomato fruit that would prove themselves by (i) showing altered nutrition value and flavor on the basis of free amino acids and saccharide content; (ii) changing the concentration of various secondary metabolites; and (iii) increasing antioxidant capacity and enzyme activities based on exposure to the abiotic (xenobiotic) form of stress.

## 2. Materials and Methods

### 2.1. Plant Growth and Fruit Harvest

Six week old tomato (*Solanum lycopersicum* L. var. ‘Cherrola‘) seedlings were grown in 4 L pots for 35 days in a vegetation hall at ambient temperature and under light and root irrigation with deionized water, as described previously [8,9]. The substrate “Horticultural Substrate with Active Humus” with added mineral nutrients (AGRO CS a.s., Říkov, Czech Republic) of 500 g D.W. (dry weight) was chosen as optimal. Every three weeks, liquid mixed fertilizer with mineral macronutrients (Lovoflor NPK 4-2.5-3, Lovochemie a.s., Lovosice, Czech Republic) was inserted into the soil at the fertilizer dose (0.75 mL·kg^−1^) optimal for nutrients supplementation.

Plants in six groups in five replicates were treated with penconazole (3.52 µmol/plant), tebuconazole (3.52 µmol/plant) or penconazole + tebuconazole (1.76 + 1.76 µmol/plant) either by foliar application or directly injected into the soil every week (i.e., five times). The seventh group served as a control. Tebuconazole (1-(4-chlorophenyl)-4,4-dimethyl-3-(1H-1,2,4-triazol-1-ylmethyl)pentan-3-ol) and penconazole (1-[2-(2,4-dichlorophenyl)pentyl]1,2,4-triazole) were of analytical-grade purity (Pestanal^®^, Sigma-Aldrich, St. Louis, MI, USA). Ripe tomato fruits were collected on the 35th day of tomato growth, immediately frozen in liquid nitrogen and stored at −80 °C until analysis. Additionally, samples of freeze-dried tomato fruit were prepared by lyophilization on Lyovac GT 2, FinnAqua, Tuusula, Finland. 

### 2.2. Determination of Free Proteinogenic Amino Acids and Total Soluble Protein Content

The content of free amino acids in freeze-dried tomato fruit was determined by capillary electrophoresis. The experiments were conducted in a fused-silica capillary (Polymicro Technologies, Phoenix, AZ, USA) using a G7100A Capillary Electrophoresis Instrument (Agilent Technologies, Waldbronn, Germany) with a contactless conductivity detector [39]. Protein content was analyzed with Bradford reagent (Sigma-Aldrich, St. Louis, USA) [40].

### 2.3. Determination of Total Soluble and Membrane-Bound Saccharides 

The tomato extracts for the measurement of total soluble and membrane-bound saccharide contents were prepared according to [41]. Briefly, freeze-dried tomato samples were ground and extracted in 50 mM Tris–HCl, 1 mM EDTA and 0.02 g/mL polyvinylpyrrolidone (PVP), pH 7.0. The homogenate was centrifuged at 20,000× *g* for 15 min at 4 °C. The resulting supernatant was used for total soluble saccharide content measurement. The pellet was resuspended in the extraction buffer containing 1 M NaCl. The suspension was centrifuged under the same conditions again, and the resulting supernatant was used for the measurement of membrane-bound (ionically bound) saccharides. The total saccharide content was measured according to the Dubois method [42].

### 2.4. Determination of Glucose, Fructose and Saccharose 

The content of glucose, fructose and saccharose in freeze-dried and peeled tomato fruits was determined by capillary electrophoresis with capacitively coupled contactless conductivity detection on the G7100A Capillary Electrophoresis Instrument (Agilent Technologies, Waldbronn, Germany) [43].

### 2.5. Total Phenolic and Flavonoid Contents and Antioxidant Capacity

Tomato peel samples were treated with 50% ethanol under continuous shaking for one hour at room temperature to extract phenolic compounds. The total content of phenolics was determined using a modified Folin–Ciocâlteu colorimetric method; antioxidant capacity was determined by the ferric ion reducing power (FRAP) assay, the trolox equivalent assay with 2,2′-azino-bis(3-ethylbenzothiazoline-6-sulfonate) (ABTS^+^) and with 2,2-diphenyl-1-picrylhydrazyl (DPPH) [44]. The total flavonoids were assayed using a modified Dowd colorimetric method [45]. 

### 2.6. Determination of Carotenoids and Chlorophylls

Samples of freeze-dried tomato fruit were extracted with pure methanol under continuous shaking for 15 min at 10 °C, modified from [46,47]. After centrifugation (Mini-Spin, Eppendorf, 13,400 RPM), absorbance at a wavelength of 470 nm, 652 nm and 665 nm was then immediately measured (Multiskan GO, Thermo Scientific, MA, USA). The content of chlorophyll *a* (C_a_), chlorophyll *b* (C_b_) and total carotenoids (C_carotenoids_) was calculated using coefficients (1, 2 and 3) by [47]:

C_a_ = 16.72A_665_ − 9.16A_652_, (1)

C_b_ = 34.09A_652_ − 15.28A_665_, (2)

C_carotenoids_ = (1000A_470_ − 1.63C_a_ − 104.96C_b_)/221, (3)

Additionally, the estimation of β-carotene (449 nm), lutein (444 nm), violaxanthin (438 nm) and neoxanthin (436 nm) contents as absorbance at appropriate wavelengths was based on [47].

### 2.7. Identification of Secondary Metabolites 

Three fresh, randomly selected, ripened fruits of each variant were peeled to extract phenolic compounds from the peel using a mixture of acetone and water (7:3). UHPLC-MS/MS analysis of the extracts was performed on a Nexera X2 UHPLC (Shimadzu, Kyoto, Japan) system coupled with a MS-8050 mass spectrometer (Shimadzu, Kyoto, Japan). Chromatographic separation was performed on a UHPLC Acquity BEH C18 (150 × 3.0 mm; 1.7 μm particle size) column (Waters Corp., Milford, MA, USA) at 40 °C. Quantification was performed using the isotope dilution method with *p*-coumaric acid-d6 and salicylic acid-d4 (Toronto Research Chemicals, Toronto, ON, Canada) as internal standards. More details were published in previous studies [9,48].

### 2.8. Antioxidant and Biotransformation Enzyme Activities

Frozen samples of tomato fruit were ground in liquid N_2_ with 1% (*w*/*v*) PVP and mixed with the extraction buffer (0.1 M Tris–HCl, pH 7.8, 1 mM EDTA, 10 mM DTT and 10 mM sodium ascorbate). The homogenate was centrifuged at 16,600× *g* for 15 min at 4 °C. The resulting supernatant was used immediately for enzyme activity measurements. The remaining sediment was washed with the same extraction buffer containing 1 M NaCl and used to measure the total concentration of bound peroxidases.

The activity of total soluble and total bound peroxidases (POX, EC 1.11.1.7) was determined using 3,3’-diaminobenzidine (DAB) as a substrate [49]. The total activity of ascorbate peroxidase (APX, EC1.11.1.11) was determined as the decrease in ascorbate absorbance at 298 nm [50]. After separation by 10% native polyacrylamide gel electrophoresis, always loading 3 µg of protein per lane, the isoenzyme pattern was detected for soluble POX using DAB [49,51], APX, superoxide dismutase (SOD, EC 1.15.1.1) and glutathione-S-transferase (GST, EC 2.5.1.18) isoforms were determined with appropriate substrates using a negative staining method with nitroblue tetrazolium [52,53] and for glutathione peroxidase (GPX, EC 1.11.1.9) 3-(4,5-dimethylthiazol-2-yl)-2,5-diphenyltetrazolium bromide was used [54]. All the chemicals were purchased at Sigma-Aldrich (USA). The approximate molecular weight was determined by the Ferguson method by performing the native electrophoretic separation under various polyacrylamide concentrations and comparing it with protein standards with known molecular weights [55].

### 2.9. Statistical Analysis

Experiments were prepared for five biological repeats. All measurements were performed in at least triplicates. Data were analyzed in SigmaPlot 12.0 (Systat Software Inc., Palo Alto, CA, USA) by one-way ANOVA (Holm–Sidak test) and t-test, with differences considered significant at *p* ≤ 0.05. Heatmaps and principal component analysis (PCA) were plotted using the Seaborn library to make statistical graphics in Python [56]. 

## 3. Results

### 3.1. Physiological Parameters and Contents of Free Amino Acids and Proteins 

**Figure 1:**
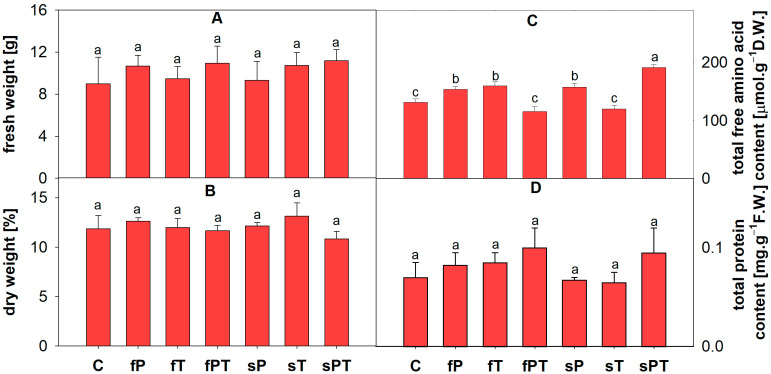
The fresh weight (**A**) and dry weight (**B**) of tomato fruit and the contents of total free proteinogenic amino acids (**C**) and soluble proteins (**D**). Penconazole (P), tebuconazole (T) or their combination (PT) were applied foliarly (fP, fT and fPT) or to the soil (sP, sT and sPT) and compared with the control treatment without triazoles (C). Different letters above each bar denote significant differences (*p* ≤ 0.05) between plant groups according to a one-way ANOVA (Holm–Sidak test). The same letters above a bar indicate that no significant differences were found between groups. Each column bar represents the mean ± SD. Experiments were prepared for five biological repeats. All measurements were performed at least three times. Abbreviations: D.W., dry weight.

The tomato fruits were collected after five times of penconazole (P), tebuconazole (T) or penconazole + tebuconazole (PT) application either foliarly (f) or directly to the soil (s) during 35 days of tomato plant growth. No significant change was found in the average fresh and dry weight of tomato fruit (Figure 1A,B). The contents of chlorophyll *a* and *b* were determined in tomato fruits, but no effect of triazole treatment was found (Figure S1). However, four groups (fP, fT, sP and sPT) significantly differed in the total free amino acid content from the control group (Figure 1C). In the total soluble protein content, the fPT and sPT groups showed slightly higher values but were not statistically significant (Figure 1D). 

**Figure 2:**
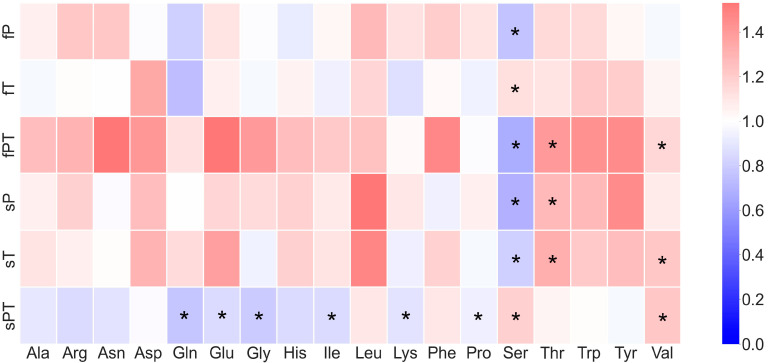
The relative content of free amino acids in tomato fruit. Penconazole (P), tebuconazole (T) or their combination (PT) were applied foliarly (fP, fT and fPT) or to the soil (sP, sT and sPT) and compared with the respective control without triazoles (C). Asterisks denote significant differences (*p* ≤ 0.05) between triazole-treated and control plants according to the *t*-test. The content of individual free amino acids was related to the total free amino acid content and then compared with the control group. Met was under the limit of detection in tomato fruits, except for the sT group, with a concentration of 0.01 ± 0.00% of total amino acids. Experiments were prepared for five biological repeats. All measurements were performed three times.

Triazole application altered the representation of the individual free amino acids, which were expressed as relative in relation to the control (Figure 2). Fruits affected by both triazoles applied to the soil (sPT) showed a significantly decreased content of Gln, Glu, Gly, Ile, Lys and Pro. On the contrary, foliar application (fPT) slightly increased the content of almost all free amino acids except Ser, Lys and Pro. The content of the precursor of phenylpropanoids, Phe, increased slightly in the fruits of some triazole-treated plants. Other aromatic amino acids, Tyr and Trp, were not significantly affected by triazoles in tomato fruits. Serine was decreased in all triazole-exposed fruits except for fT and sPT (Figure 2). All triazole-treated groups showed significantly different content of Ser, which decreased in the fP, fPT, sP and sT groups and increased in the fT and sPT groups.

### 3.2. Saccharide Contents 

**Figure 3:**
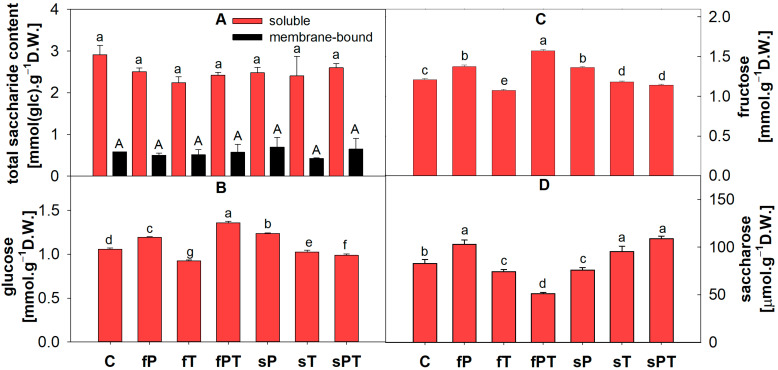
Total soluble and membrane (ionically bound) saccharide contents (**A**), glucose (**B**), fructose (**C**) and saccharose (**D**) in tomato fruits. Penconazole (P), tebuconazole (T) or their combination (PT) were applied foliarly (fP, fT and fPT) or to the soil (sP, sT and sPT) and compared with a control without triazoles (C). Different letters above each bar denote significant differences (*p* ≤ 0.05) between plant groups according to a one-way ANOVA (Holm–Sidak test). The same letters above a bar indicate that no significant differences were found between groups. Each column bar represents the mean ± SD. Experiments were prepared for five biological repeats. All measurements were performed on at least three times. Abbreviations: D.W., dry weight; F.W., fresh weight; glc, glucose.

In tomato fruits, total soluble and membrane (ionically bound) saccharide contents did not show any statistically significant changes (Figure 3A). However, a slight decrease in the total soluble saccharide content in the triazole-treated groups in comparison with the control was observed (Figure 3A). The statistically highest content of monosaccharides glucose and fructose was found in the fPT group, which correlates with the lowest saccharose content, indicating cleavage of saccharose in these plants (Figure 3). On the contrary, the lowest content of glucose and fructose was found in fT plants (Figure 3B,C). The groups fP and sPT were rich in saccharose (Figure 3D). 

### 3.3. Total Phenolics, Flavonoids, Carotenoids and Antioxidant Capacity 

**Figure 4:**
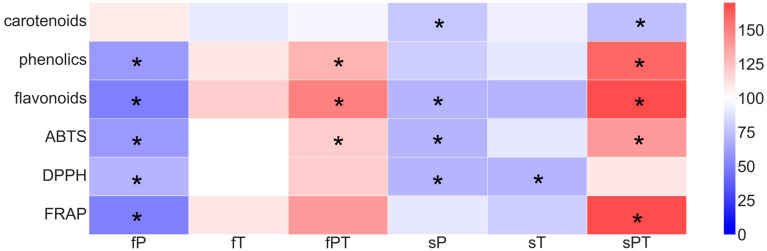
Total carotenoids, phenolics, flavonoids and antioxidant capacity measured using three different methods (ABTS, DPPH and FRAP) in tomato fruit. Penconazole (P), tebuconazole (T) or their combination (PT) were applied foliarly (fP, fT and fPT) or to the soil (sP, sT and sPT) and compared with a control without triazoles. Values are expressed as % of control (C). Asterisks denote significant differences (*p* ≤ 0.05) between the treated and control plants according to the one-way ANOVA (Holm–Sidak test). Experiments were prepared for five biological repeats. All measurements were performed at least three times.

Total phenolics and flavonoids were correlated with antioxidant capacity using two radical quenching-based methods (DPPH and ABTS methods) and the FRAP method. Applying triazole combinations, i.e., fPT and sPT, significantly increased the content of total phenolics and flavonoids and the antioxidant capacity, as measured by ABTS (Figure 4), in tomato fruits. The application with a single triazole (fP, sP and sT) showed decreases in all these parameters, with significant reductions in the fP group. Only the fT group did not differ from the control tomato fruits. Total carotenoid content was significantly lower in the sP and sPT groups than in the control group (Figure 4). Several individual carotenoids were measured spectrophotometrically (Figure S2).

### 3.4. Identification of Secondary Metabolites in Tomato Peel

**Figure 5:**
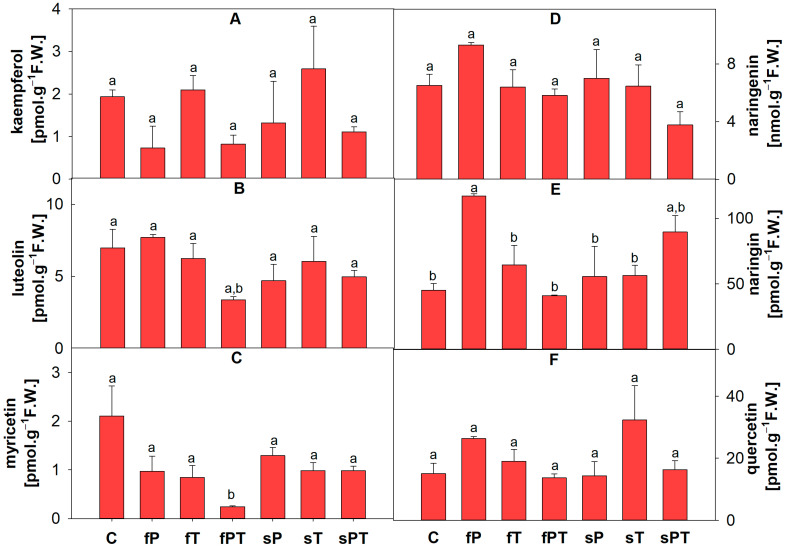
Identification and content of flavonoids in the peel of tomato fruits based on appropriate standards: kaempferol (**A**), luteolin (**B**), myricetin (**C**), naringenin (**D**), naringin (**E**), and quercetin (**F**). Penconazole (P), tebuconazole (T) or their combination (PT) were applied foliarly (fP, fT and fPT) or to the soil (sP, sT and sPT) and compared with the respective control without triazoles (C). Different letters above each bar denote significant differences (*p* ≤ 0.05) between plant groups according to a one-way analysis of variance (ANOVA; Holm–Sidak test). The same letters above a bar indicate that no significant differences were found between groups. Each column bar represents the mean ± SD. Experiments were prepared for five biological repeats. All measurements were performed at least three times. Abbreviations: F.W., fresh weight.

**Figure 6:**
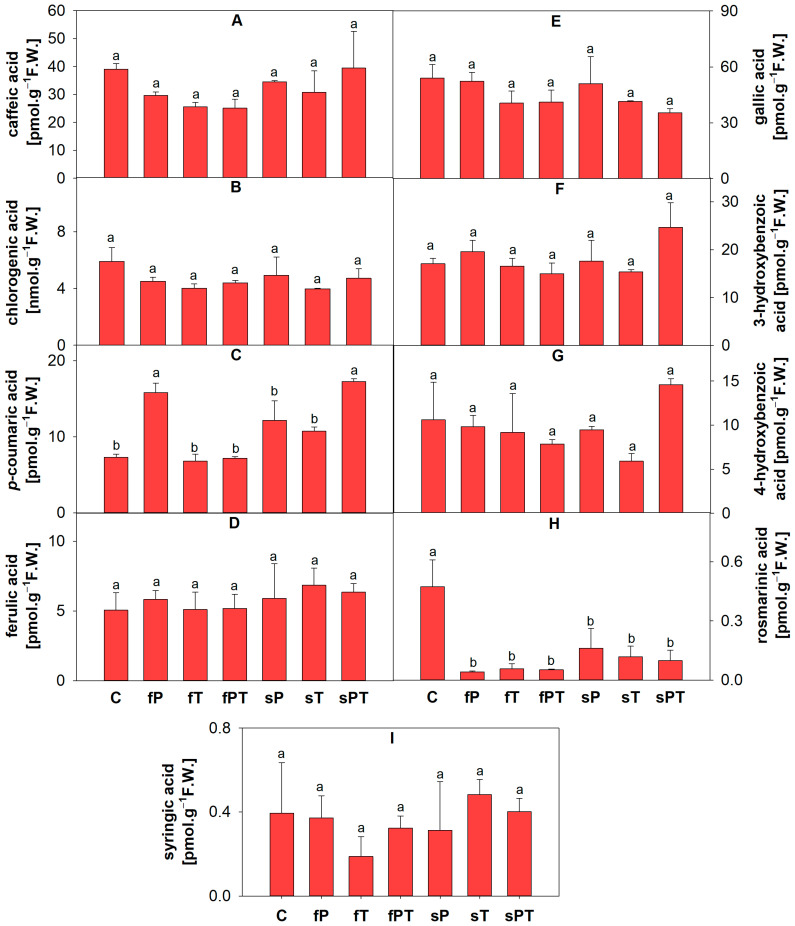
Identification and content of phenolic acids in the peel of tomato fruits based on appropriate standards: caffeic acid (**A**), chlorogenic acid (**B**), *p*-coumaric acid (**C**), ferulic acid (**D**), gallic acid (**E**), 3-hydroxybenzoic acid (**F**), 4-hydroxybenzoic acid (**G**), rosmarinic acid (**H**), and syringic acid (**I**). Penconazole (P), tebuconazole (T) or their combination (PT) were applied foliarly (fP, fT and fPT) or to the soil (sP, sT and sPT) and compared with the respective control without triazoles (C). Different letters above each bar denote significant differences (*p* ≤ 0.05) between plant groups according to a one-way analysis of variance (ANOVA; Holm–Sidak test). The same letters above a bar indicate that no significant differences were found between groups. Each column bar represents the mean ± SD. Experiments were prepared for five biological repeats. All measurements were performed at least three times. Abbreviations: F.W., fresh weight.

**Figure 7:**
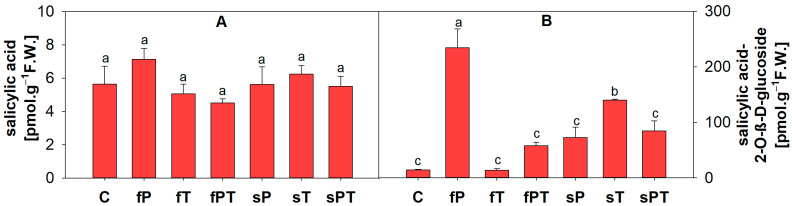
The content of salicylic acid (**A**) and salicylic acid 2-O-β-D-glucoside (**B**) in the peel of tomato fruits is based on appropriate standards. Penconazole (P), tebuconazole (T) or their combination (PT) were applied foliarly (fP, fT and fPT) or to the soil (sP, sT and sPT) and compared with the respective control without triazoles (C). Different letters above each bar denote significant differences (*p* ≤ 0.05) between plant groups according to a one-way analysis of variance (ANOVA; Holm–Sidak test). The same letters above a bar indicate that no significant differences were found between groups. Experiments were prepared for five biological repeats. All measurements were performed at least three times. Each column bar represents the mean ± SD. Abbreviations: F.W., fresh weight.

To assess whether triazoles alter the content of flavonoids and phenolic acids in tomato peel, seventeen secondary metabolites were identified in tomato peels by LC-MS, based on their appropriate standards, and expressed per g of fresh tomato peel weight (Figure 5, Figure 6 and Figure 7). 

Among the monitored phenylpropanoids, the most abundant was naringenin (Figure 5D), followed by chlorogenic acid (Figure 6B). The highest naringenin content as well as naringin content (Figure 5E) was found in the fP group. In turn, the fPT group showed the lowest contents of the flavonoids naringin, kaempferol, luteolin and myricetin (Figure 5A–C).

The concentration of chlorogenic acid was slightly lower in all triazole-treated groups than in the control tomato fruits (Figure 6B). The fP and sPT groups stood out statistically significantly in the high content of *p*-coumaric acid (Figure 6C). All triazole-treated groups showed a significantly lower content of rosmarinic acid in comparison with the control (Figure 6H). When penconazole and tebuconazole were co-administered, the tomatoes showed the effect of the application method; in other words, the concentrations of luteolin, naringin, 3- or 4-hydroxybenzoic acid, and rosmarinic acid were slightly higher, and myricetin and *p*-coumaric acid were significantly higher in soil drenching than in spraying treatments (Figure 5 and Figure 6).

Salicylic acid is also a plant phytohormone. The fP group showed not only a slight increase in the salicylic acid content in comparison with the control (Figure 7A) but also a statistically significant increase in the content of its metabolite, salicylic acid 2-O-β-D-glucoside (Figure 7B). Except for the fT group, all the triazole-treated groups had a higher content of salicylic acid 2-O-β-D-glucoside (Figure 7B).

### 3.5. Activites and Isoforms of Antioxidant Enzymes 

**Figure 8:**
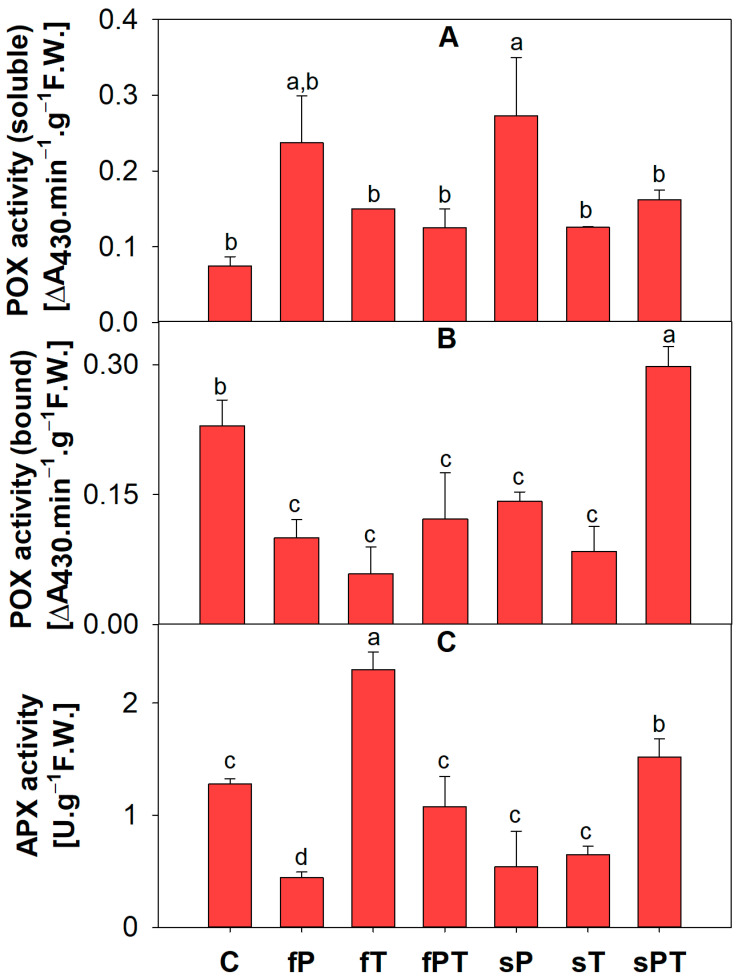
Fresh weight activity of soluble peroxidases (**A**), membrane-bound peroxidases (**B**) and ascorbate peroxidase (**C**) in tomato fruits. Penconazole (P), tebuconazole (T) or their combination (PT) were applied foliarly (fP, fT and fPT) or to the soil (sP, sT and sPT) and compared with a control without triazoles. Different letters above each bar denote significant differences (*p* ≤ 0.05) between plant groups according to a one-way analysis of variance (ANOVA; Holm–Sidak test). The same letters above a bar indicate that no significant differences were found between groups. Each column bar represents the mean ± SD. Experiments were prepared for five biological repeats. All measurements were performed at least three times. Abbreviations: APX, ascorbate peroxidase; POX, peroxidases; F.W., fresh weight.

The activity of soluble peroxidases (POX) was significantly higher in the sP group than in the control tomato fruits, followed by the fP group (Figure 8A). As for the membrane-bound POX, all triazole-treated groups showed significantly lower activity than the control, except for the sPT group, which was significantly higher (Figure 8B). The activity of ascorbate peroxidase (APX) was significantly higher in the fT and sPT groups than in the control group. Other triazole-treated groups showed a significant decrease in APX activity in comparison with the control (Figure 8C).

**Figure 9:**
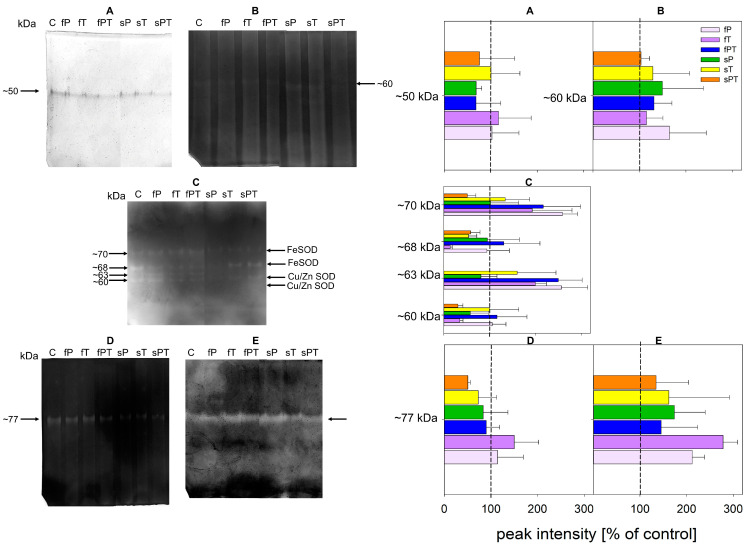
Tomato fruit isoforms of soluble peroxidases (**A**), ascorbate peroxidase (**B**), superoxide dismutase (**C**), glutathione-S-transferase (**D**) and glutathione peroxidase (**E**) detected after native electrophoresis in 10% polyacrylamide gels with the appropriate densitometric evaluation of the relative intensity of the bands represented by a bar graph (corresponding (**A–E**) on the right side). The same amount of proteins (3 µg) was applied to each line. Inhibition tests of SOD isoforms with H_2_O_2_ and KCN (not shown) enabled the identification of particular isoforms, similarly as analysis in five various polyacrylamide gel concentrations (5–10%) with appropriate native protein standards (not shown) enabled relative weight estimation by the Ferguson method.

In tomato fruits, two POX isoforms were detected, namely a 50 kDa (Figure 9A) isoform that was not affected by triazoles. Only one APX isoform remained unaffected by triazoles in fruits (Figure 9B). We detected four isoforms of SOD at 70, 68, 63 and 60 kDa (Figure 9C). The inhibition tests of SOD isoenzymes with H_2_O_2_ and KCN indicated that the ~70, and ~68 kDa SOD isoforms were FeSOD enzymes, whereas the ~60 and 63 kDa isoforms were Cu/ZnSOD enzymes. All foliar triazole applications (fP, fT and fPT) increased the activity of the ~70 and ~63 kDa SOD isoforms (Figure 9C). One ~77 kDa GST isoform was detected, which did not respond to the treatment with triazoles (Figure 9D), as well as only one GPX isoform (Figure 9E). 

**Figure 10:**
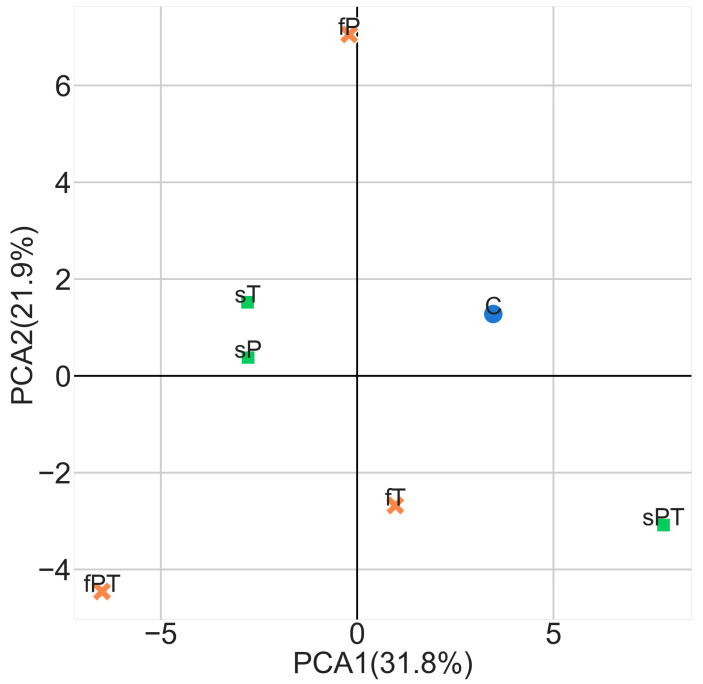
PCA analysis of all tomato fruit experimental groups based on the results of all measurements. Penconazole (P), tebuconazole (T) or their combination (PT) were applied foliarly (fP, fT, fPT and orange crosses) or into the soil (sP, sT, sPT and green squares). In the control group, triazoles were substituted with deionized water (C, blue circle).

We subjected all experimental data to principal component analysis (PCA) to determine how far the application of triazoles, either in soil or by spraying, affects tomato fruit overall, i.e., how much it differs from the untreated control. PCA showed that the fPT group was negatively correlated with the control based on all experimental measurements (Figure 10). While the groups sT and sP clustered together, they negatively correlated with the sPT. The foliar application of penconazole (fP), tebuconazole (fT) and combination (fPT) did not cluster together, insinuating a different impact on the tomato fruit for each variant.

## 4. Discussion

Triazole fungicides are widely used in agriculture for the protection of crop yields [8,9]. Here, the effect of penconazole, tebuconazole and their combination, applied directly to the soil or foliarly on *Solanum lycopersicum* L. (cv. ‘Cherrola’) plants, on the nutritional quality of fruit was determined by the content of saccharides, free amino acids and flavonoids, and monitoring the antioxidant enzymatic and non-enzymatic systems. The quality of the tomato fruit depends on its firmness and other physical properties, carbohydrate content, organic acids, and other nutritionally valuable substances such as free amino acids and proteins. Other substances with antioxidant capacity, especially vitamin C and carotenoids, including lycopene, are also beneficial to the consumer. Tomatoes contain several secondary metabolites such as flavonoids, phenolic acids and terpenoids, including volatile compounds. These compounds also have antioxidant properties, in addition to other health benefits [33,57,58,59].

No significant impact of triazoles was observed on the fresh weight of fruit (Figure 1A). After foliar treatment of tomato plants with various combinations of cyproconazole, penconazole and tebuconazole, Jakl et al. determined differences in the fruit fresh weight in the first earlier harvest but then a return to similar values in the late second harvest comparable with this article [8].

Several metabolic changes occur in maturing fruit: photosynthesis is significantly reduced, organic acids are converted into carbohydrates, and carotenoids are synthesized at an increased rate [57]. These processes were also affected by applying triazoles, as evidenced by differences in the total free amino acid content of tomato fruits (Figure 1C). The decrease in Glu content in sPT could be associated with the synthesis of Pro (Figure 2) and carbohydrates (Figure 3) or with the downregulation of chlorophyll synthesis. The synthesis of phenolic compounds is also likely to occur in fruits; a precursor of these metabolites, Phe, was slightly increased in the fruits of triazole-treated plants, but other aromatic amino acids, such as Tyr and Trp, were not significantly affected by triazoles (Figure 2). Additionally, the tomato fruit did not significantly differ in the soluble protein content, but the slight increase in the fPT and sPT groups could insinuate a response to the triazole combination treatment in, e.g., proteosynthesis of antioxidant enzymes (Figure 1D). The decarboxylation of Ser leads to the formation of ethanolamine, which is a building block for plant membrane phospholipids as well as a signaling molecule [60]. Therefore, the significant decrease in Ser content in the triazole-treated groups (Figure 2) may be related to modifications of the cell membrane or cell wall in order to restrict the triazole uptake.

Saccharose is the major form of carbohydrates transported to fruits from the photosynthetic organs. During ripening, saccharose is either hydrolyzed by invertase into glucose and fructose or split by saccharose synthase into fructose and uridine diphosphate- (UDP-) glucose while using UDP [61]. The fPT group showed significantly decreased saccharose content (Figure 3D), i.e., higher concentrations of glucose and fructose that improve tomato flavor for consumers.

In tomato fruits, flavonoids accumulate in the peel, with only traces found in the flesh. The most abundant flavonoids are naringenin and various sugar conjugates of the flavonols quercetin and kaempferol, including rutin, while the major phenolics in tomatoes are chlorogenic and caffeic acids [57]. Triazole treatment seems to alter the content of various single secondary metabolites or their biosynthetic pathways, as was also reported in other studies [8,9]. The combination of penconazole and tebuconazole applied directly to the soil or foliarly (fPT and sPT) significantly increased the total phenolic and flavonoid contents (Figure 4). However, the representation of individual secondary metabolites (based on the used standards) slightly differed between the triazole-treated and control groups. Tomato fruits contain a vast array of phenolics and flavonoids [4,57]. Therefore, the difference (Figure S3) in the measured content of total phenolic acids and flavonoids in comparison with individual identified compounds (based on 17 standards) may be caused by other non-detected secondary metabolites, which can have a significant impact on antioxidant properties. The fPT group showed the significantly lowest content of flavonoids luteolin, myricetin and rosmarinic acid in comparison with the control (Figure 5B,C and Figure 6H). All triazole-treated plants showed a significantly decreased content of rosmarinic acid in the fruit (Figure 6H). It was shown that postharvest treatment with rosmarinic acid delayed the ripening of tomato fruits [61]. Here, especially the application of the combination of penconazole and tebuconazole could have sped up the ripening process, as indicated by the low concentration of rosmarinic acid (Figure 6H), significantly higher amounts of fructose and glucose for fPT (Figure 3B,C), and significantly increased total phenolics and flavonoids for both fPT and sPT (Figure 4) than the control. Difenoconazole was also shown to increase the content of both total phenolics and flavonoids, as well as carotenoids [62]. 

Diet is generally considered to be a key determinant of human health. It is often recommended to increase the diversity and nutrient density of the foods consumed and to reduce the intake of compounds known to increase the risk of disease. Such restrictions involve limiting the intake of certain types of carbohydrates and fat [63]. On the other hand, the increase of health-beneficial compounds such as flavonoids is generally welcomed since several attributes involving antioxidant, anti-inflammatory, anti-mutagenic and anti-carcinogenic properties have been reported [64]. The total phenolics and flavonoids were significantly higher in the groups treated with both triazoles, regardless of the application form (Figure 4). The higher contents of these secondary metabolites increase the quality of tomato fruit for consumers, but the potential risk of triazole residues must also be considered. Apart from carbohydrates and fats, the other macronutrient, protein, is often considered an invariable constant. However, each amino acid has its own specific metabolism and is important for numerous cellular and physiological processes [63]. It has been shown that variation in dietary intake of amino acids such as serine, glycine, asparagine, histidine and methionine influences health and disease, including cancer, through defined molecular mechanisms [63]. Except for the fT and sPT groups, the serine content was significantly lower in the triazole-treated groups than in the control (Figure 2). Although serine is a non-essential amino acid, its dietary uptake restriction was related to reduced tumor growth [65,66]. Therefore, its low content in the tomato fruit could be desirable for certain consumers; however, the triazole residues in the fruit may offset this possible advantage.

Except for the increased ethylene production, tomato fruit ripening is also associated with increased respiration rates, thus the production of reactive oxygen species [61,62]. Therefore, both non-enzymatic and enzymatic parts of the antioxidant system play a key role in fruit maturation. Foliar application of penconazole (fP) significantly increased the activity of total soluble peroxidases, while the activity of total membrane-bound peroxidases and ascorbate peroxidase decreased in comparison with the control (Figure 8). Additionally, the fP group significantly decreased all parameters of the non-enzymatic antioxidant system (Figure 4), while significantly increasing the contents of naringin (Figure 5E), *p*-coumaric acid (Figure 6C), and salicylic acid 2-O-β-D-glucoside (Figure 7B). Naringin is a glycosylated form of naringenin. Both have been regarded as having antioxidant and health-beneficial properties [67]. The high concentration of naringin may indicate a response to oxidative stress caused by the foliar application of penconazole, but at the same time, its higher content is advantageous for consumers. *p*-Coumaric acid is one of the precursors of lignin, a basic building block of the plant cell wall [68]. The increase in its concentration may be related to the cell wall strengthening in order to limit the entry of triazoles (as xenobiotics) into the plant tissue. Salicylic acid 2-O-β-D-glucoside is a key metabolite of salicylic acid (a phytohormone), but it also works as an antioxidant. The external treatment of *Arabidopsis* with salicylic acid 2-O-β-D-glucoside led to a significant reduction of bacterial infection by *Pseudomonas syringae* [69]. It seems that this compound has a good potential to be a non-toxic plant protectant against various stresses [69]. Thus, we may conclude that the significant increase in salicylic acid 2-O-β-D-glucoside concentration in the fP group may relate to abiotic stress caused by penconazole (Figure 7B). Apart from SOD, all the detected antioxidant enzyme isoforms after electrophoretic separation did not respond to the triazole treatment (Figure 9). The foliar triazole applications (fP, fT and fPT) increased the activity of the ~70 and ~63 kDa SOD isoforms (Figure 9C). SODs are crucial antioxidant enzymes, protecting plants against biotic and abiotic stress. At least nine SOD genes exist in tomatoes, including four Cu/ZnSODs, three FeSODs and one MnSOD; some of them are expressed only in young fruits [70]. The increase in SOD activity under triazole application was observed together with severe abiotic stresses [71,72,73,74,75]. Here, we may assume that the foliar triazole treatment directly acts on tomato fruit and SOD as the first line of antioxidant defense response to the abiotic (oxidative) stress caused by triazoles.

From the PCA analysis (Figure 10), we can conclude that the application of penconazole and tebuconazole together leads to more differences in the tomato fruit parameters in comparison with the control than the single treatments. It is also interesting to point out that almost no difference was calculated between the soil application of single penconazole and tebuconazole, while the foliar treatment of these triazoles affects the tomato fruit differently.

In the Pearson’s correlation analysis of all triazole-treated groups (Figure S3), the secondary metabolites 3-hydroxybenzoic acid, 4-hydroxybenzoic acid, caffeic acid and chlorogenic acid, we found a positive correlation with free amino acids, especially Asn, Asp, Gln, Glu, Lys and Phe, as well as a positive correlation with the total free amino acids and the content of membrane-bound saccharides (Figure S3). On the other hand, there was a rather negative correlation between quercetin, syringic acid, kaempferol, and ferulic acid and several free amino acids (Figure 3). Free amino acids are involved in plant development and response to environmental stressors, as well as serving as precursors for many primary and secondary metabolites. In addition, they have a pivotal role in human nutrition [76]. For tomato fruit consumption, there must most likely be a compromise between the presence of free amino acids, or phenolics, and flavonoids (i.e., antioxidants). Total membrane-bound saccharides positively correlated with the content of chlorogenic acid (0.89), and the activity of membrane-bound peroxidases correlated with the chlorogenic acid (0.63) and total soluble (0.81) as well as membrane-bound saccharides (0.65), which is most likely related to the cell wall strengthening (Figure S3). Rosmarinic acid showed a negative correlation with both total and individual carotenoids (Figure S3). However, our identified correlations do not establish causation since further experiments and investigations for confirmation are required. 

Even though the plant detoxification enzyme (GST, Figure 9D) and the antioxidant enzyme system (Figure 9) were mostly not induced by applying triazoles and some metabolic changes can be considered positive (Figure 3, Figure 4, Figure 6H and Figure 7B), the prevalence of residual triazoles in edible fruits may still harm consumers (especially if eating unwashed fruits). Kovač et al. showed that some triazoles remained in tomato fruit peels in concentrations above their permissible limits [34]. Moreover, high concentrations of triazoles can cause endocrine disorders by interfering with human cytochromes in steroid hormone biosynthesis [77,78].

## 5. Conclusions

Antifungal triazoles can both protect the tomato yield against fungal infection but also influence the quality of the tomato fruit’s nutrition as a side effect. Although the content of carbohydrates, proteins and total free amino acids in tomato fruit was not much affected by the application of triazoles, the representation of individual amino acids varied, with a particularly marked reduction in serine. There was a difference in the relative increase in several amino acids after foliar spraying of the triazole combination, while there was a decrease after soil application. The equimolar combination of penconazole and tebuconazole applied directly to the soil or foliarly affected most of the nutrition parameters in comparison with the control, especially positively impacting the total phenolic and flavonoid contents as well as antioxidant capacity. In turn, foliar application of penconazole seemed to represent the most stressful treatment for the tomato fruits, showing a significant decrease in all parameters of the non-enzymatic antioxidant system.

## Data Availability

Data are contained within the article or supplementary material. The raw data are available on request from the corresponding author.

## Supplementary Materials

The following supporting information can be downloaded at: https://www.mdpi.com/article/10.3390/metabo13090988/s1, Figure S1. Contents of chlorophyll *a* (A) and chlorophyll *b* (B). Figure S2. Contents of β-carotene (absorbance at 449 nm), lutein (444 nm), lycopene (502 nm), neoxanthin (436 nm) and violaxanthin (438 nm) in comparison with the control group without any triazole application. Figure S3. Heatmap showing Pearson’s correlation coefficients among all measurements in the triazole-treated groups.
