# Supplementary material for: Triazoles as a Potential Threat to the Nutritional Quality of Tomato Fruits
Source: Metabolites. 2023 Sep 1;13(9):988. doi: 10.3390/metabo13090988 (PMC10536328; doi:10.3390/metabo13090988)
Supplement: Supplementary file 1 [file metabolites-13-00988-s001.zip › metabolites-2574901-supplementary.pdf]

Supplement

Triazoles as a potential threat to the nutrition quality of tomato fruits

Veronika Hýsková[1], Michal Jakl[2], Jana Jaklová Dytrtová[3], Sanja Ćavar Zeljković[4,5], Ondřej Vrobel[4,5], Kateřina Bělonožníková[1], Daniel Kavan[1], Tomáš Křížek[6], Alice Šimonová[6], Marie Vašková[1], Ishak Kovač[3], Antoniana Račko Žufić[1] and Helena Ryšlavá[1*]

[1] Faculty of Science, Department of Biochemistry, Charles University, Albertov 6, 128 00 Praha 2, Czech Republic; veronika.hyskova@natur.cuni.cz, katerina.belonoznikova@natur.cuni.cz, daniel.kavan@natur.cuni.cz, ouskovam@natur.cuni.cz, zuficnina@gmail.com, helena.ryslava@natur.cuni.cz

[2] Faculty of Agrobiology, Food and Natural Resources, Department of Agroenvironmental Chemistry and Plant Nutrition, Czech University of Life Sciences Prague, Kamýcká 129, 165 00 Praha - Suchdol, Czech Republic; jakl@af.czu.cz

[3] Faculty of Physical Education and Sport, Sport Sciences—Biomedical Department, Charles University, José Martího 269, 162 52 Praha 6, Czech Republic; dytrtova@ftvs.cuni.cz, ikovac@ftvs.cuni.cz

[4] Centre of the Region Haná for Biotechnological and Agricultural Research, Department of Genetic Resources for Vegetables, Medicinal and Special Plants, Crop Research Institute, Šlechtitelů 241/27, 783 71 Olomouc - Holice, Czech Republic; sanja.cavar@upol.cz, ondrej.vrobel@upol.cz

[5] Czech Advanced Technology and Research Institute, Palacký University, Křížkovského 511/8, 779 00 Olomouc, Czech Republic; sanja.cavar@upol.cz, ondrej.vrobel@upol.cz

[6] Faculty of Science, Department of Analytical Chemistry, Charles University, Albertov 6, 128 00 Praha 2, Czech Republic; tomas.krizek@natur.cuni.cz, alice.simonova@natur.cuni.cz

* Correspondence: helena.ryslava@natur.cuni.cz; Tel.: +420 221 951 282

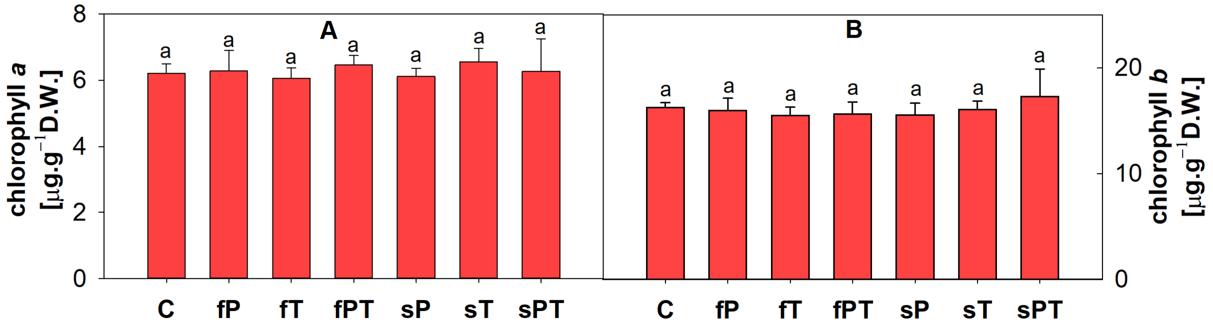

Figure S1. Contents of chlorophyll *a* (A) and chlorophyll *b* (B). Different letters above each bar denote significant differences ($p \leq 0.05$) between plant groups according to a one-way analysis of variance (ANOVA; Holm-Sidak test). The same letters above a bar indicate that no significant differences were found between groups. Each column bar represents the mean ± SD. Abbreviations: D.W., dry weight.

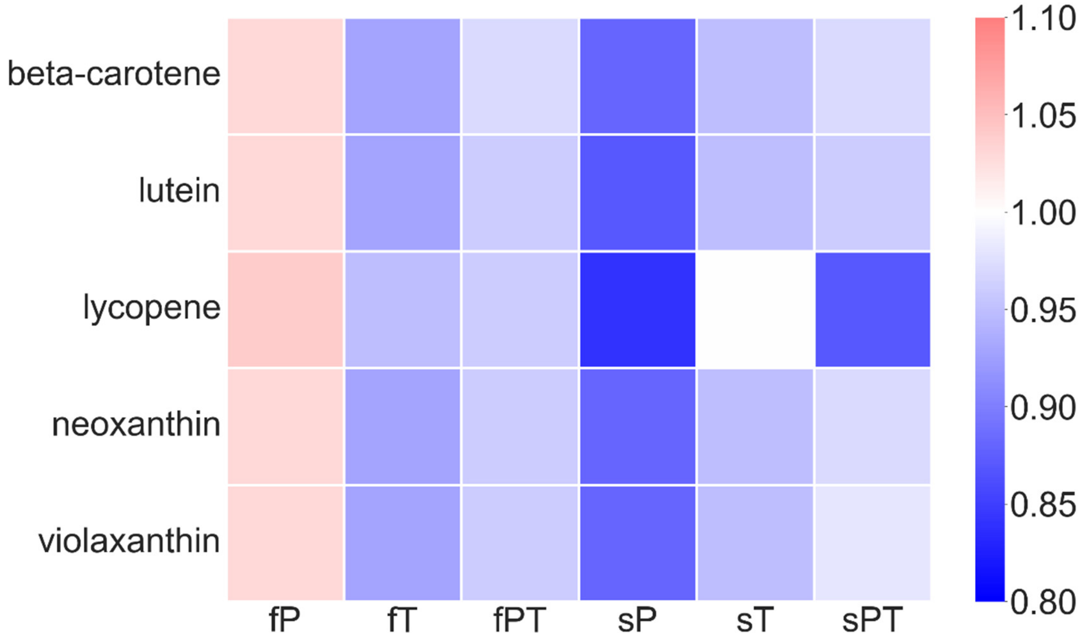

Figure S2. Contents of β-carotene (absorbance at 449 nm), lutein (444 nm), lycopene (502 nm), neoxanthin (436 nm), and violaxanthin (438 nm) in comparison with the control group without any triazole-application.

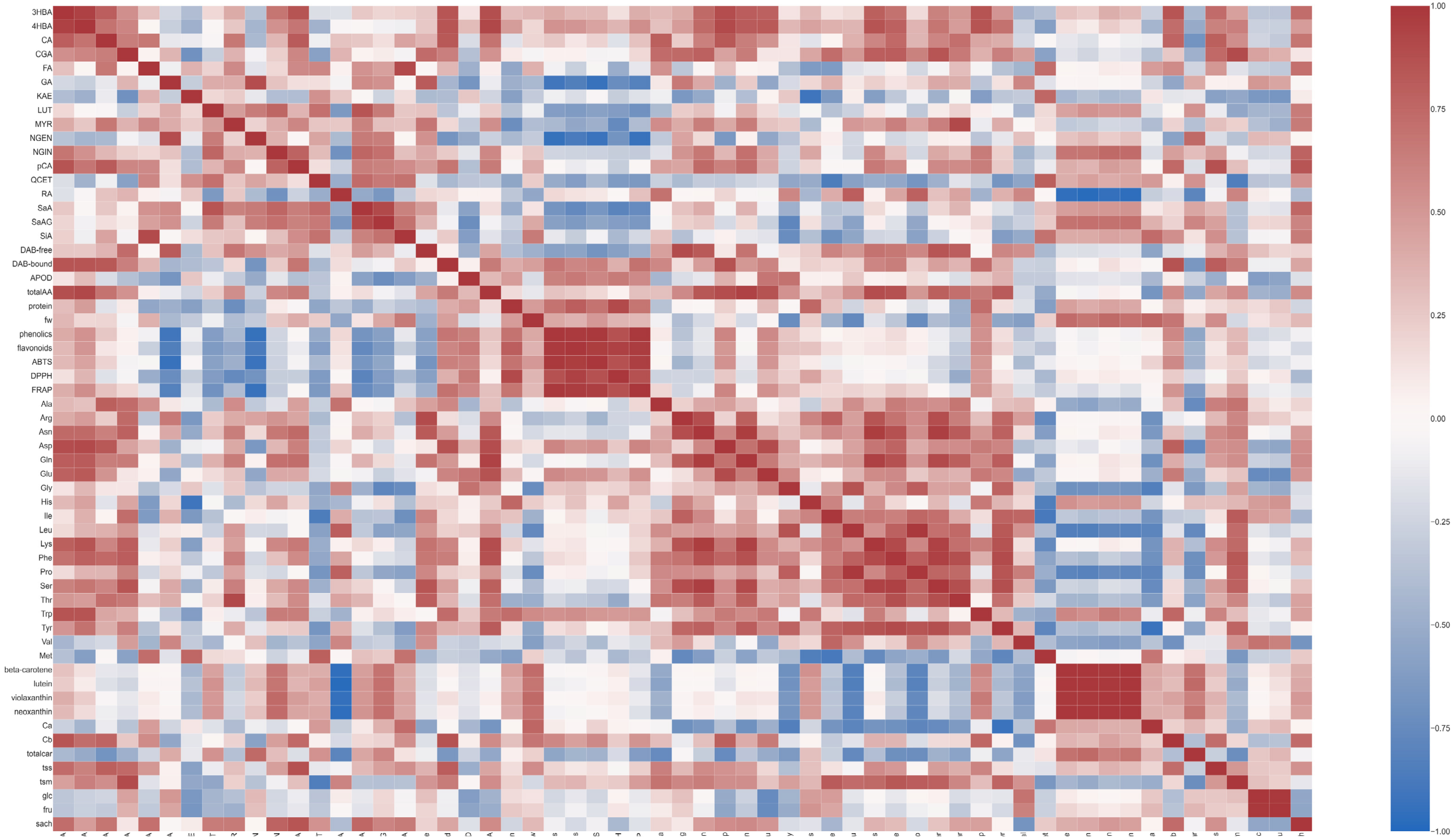

Figure S3. Heatmap showing Pearson's correlation coefficients among all measurements in the triazole treated groups. Abbreviations: 3HBA, 3-hydroxybenzoic acid; 4HBA, 4-hydroxybenzoic acid; CA, caffeic acid; CGA, chlorogenic acid; FA, ferulic acid; GA, gallic acid; KAE, kaempferol; LUT, luteolin; MYR, myricetin; NGEN, naringenin; NGIN,

naringin; pCA, *p*-coumaric acid; QCET, quercetin, RA, rosmarinic acid; SaA, salicylic acid, SaAG, salicylic acid 2-O-β-D-glucoside, SiA, syringic acid; DAB-free, total soluble peroxidases; DAB-bound, total membrane-bound peroxidases; APOD, ascorbate peroxidase; total AA, total amino acid content; protein, total protein content; fw, fresh weight; phenolics, total phenolic content; flavonoids, total flavonoid content; ABTS, antioxidant capacity measured by ABTS method; DPPH, antioxidant capacity measured by DPPH method; FRAP, antioxidant capacity measured by FRAP method; Ca, chlorophyll a; Cb chlorophyll b; totalcar, total carotenoids; tss, total soluble saccharides; tsm, total membrane-bound saccharides; glc, glucose; fru, fructose; sach, saccharose.
